# Longer-Term Effects of the Glycaemic Index on Substrate Metabolism and Performance in Endurance Athletes

**DOI:** 10.3390/nu15133028

**Published:** 2023-07-04

**Authors:** Anna Maria Moitzi, Daniel König

**Affiliations:** 1 Division for Nutrition, Exercise and Health, Department of Nutritional Sciences, Faculty of Life Sciences, University of Vienna, 1090 Vienna, Austria; anna.moitzi@univie.ac.at; 2 Vienna Doctoral School of Pharmaceutical, Nutritional and Sport Sciences, University of Vienna, 1090 Vienna, Austria; 3 Division for Nutrition, Exercise and Health, Department of Sport Science, Centre for Sports Science and University Sports, University of Vienna, 1150 Vienna, Austria

**Keywords:** glycaemic index, long-term effects, sport nutrition, endurance performance, substrate metabolism

## Abstract

Nutrition has a decisive influence on athletic performance. However, it is not only the nutrient intake during exercise that is important, but the daily diet must also be adapted to the requirements of physical activity in order to optimally promote training adaptations. The goal of prolonged endurance training is to enhance fat oxidation, to maintain aerobic performance at a higher intensity while sparing limited carbohydrate stores. The targeted modification of macronutrient intake is a common method of influencing substrate metabolism, fuel selection, and performance. However, it is not well established whether the glycaemic index of carbohydrates in our daily diet can improve endurance performance by influencing carbohydrate or fat oxidation during training. Therefore, the aim of the following review is to elucidate the possible influence of the glycaemic index on substrate utilization during exercise and to clarify whether the consumption of a long-term high-carbohydrate diet with different glycaemic indices may have an influence on substrate metabolism and endurance performance.

## 1. Introduction

Endurance athletes should not only pay attention to sufficient carbohydrate intake during exercise, but also to the carbohydrate content of their daily diet, in order to optimally replenish glycogen stores. The recommended amount of daily CHO intake depends on several factors, including training frequency, duration, and intensity. Therefore, the recommendation for CHO intake varies from 3 to 12 g·kg body weight^−1^·day^−1^. Depending on which metabolic or structural adaptation is to be the focus of the current training load, it must be decided whether more or less carbohydrates should be supplied [1,2]. 

Furthermore, it is important for athletes to be mindful of the nutritional and physiological effects associated with their dietary intake. The quality of carbohydrates, and in particular the glycaemic index, can significantly influence the metabolic processes after ingestion. The glycaemic index (GI) categorizes carbohydrates according to their impact on blood glucose concentration and the extent to which they stimulate insulin secretion, usually compared to glucose (GI = 100) or white bread (GI = 70) [3,4]. Therefore, the GI reflects the availability of the consumed CHO in the blood. So, for the same amount of carbohydrates, a low-GI food (GI ≤ 55) will not raise blood glucose to the same extent as a high-GI food (GI ≥ 70). Low-GI foods are therefore digested and absorbed more slowly compared to high-GI foods [5,6]. Among other factors, the relative ratio of carbohydrate to fat oxidation is decisively co-regulated by the level of insulin concentration in the blood. The higher the GI, the faster the blood glucose rises, the higher the insulin secretion, and the lower the fat oxidation. This relationship has already been demonstrated in athletes during endurance activity [7,8].

For improving performance, an athlete needs a combination of adequate fuel stores to provide enough ATP for muscle work and metabolic flexibility. Metabolic flexibility refers to the capacity to effectively utilize various pathways to optimize ATP regeneration, while also enabling the utilization of all muscle fuel sources to meet the specific demands of exercise at different intensities [9]. So far, it has been believed that a low-carbohydrate diet has beneficial effects on fat oxidation. However, the disadvantage of these so-called low-carbohydrate diets is that performance at higher intensities deteriorates because metabolic flexibility is worsened by the absence of carbohydrates [10]. In theory, with the help of the GI and its influence on postprandial insulin secretion and fat oxidation, it should be possible to improve metabolic flexibility by improving fat oxidation and having sufficient carbohydrates for energy provision at high intensities. 

The present review aims to investigate whether the glycaemic index plays a role in endurance sports and if the consumption of long-term carbohydrate-rich diets with different glycaemic indices can have an influence on substrate metabolism and endurance performance.

### 1.1. Mechanism of Substrate Oxidation during Endurance Exercise and Influencing Factors

During prolonged endurance exercise, the main source of ATP fuel for muscle work is derived from the oxidative phosphorylation of both fat and carbohydrates (CHO). The primary substrates utilized by the muscles, include muscle and liver glycogen, blood glucose, and fatty acids derived from muscle and adipose tissue triglyceride stores [11,12]. The oxidation of proteins for energy production is less important compared to the primary sources—carbohydrates and fats—and they contribute only about 5% to ATP supply [12]. As the intensity of exercise increases, there is a greater reliance on glucose and glycogen as fuel sources, surpassing the oxidation of fat. This shift occurs because carbohydrates provide a greater energy output per unit of time, leading to an increased reliance on glucose and glycogen [13,14,15]. In healthy individuals, maximal rates of fat oxidation can be expected at intensities of 60–65% of maximal oxygen uptake (VO_2_max) [16] and vary from 0.18 to 1.01 g·min^−1^ [17]. With glycogen stores in the muscles and liver limited to approximately 2000 kcal, they represent one of the most important limiting factors for prolonged endurance exercise at higher intensities. Research over recent decades has shown that the most effective diet is the one that is able to augment and preserve CHO fuel stores (i.e., muscle and liver glycogen) for the decisive phases of a race [18]. Fat stores, on the other hand, are normally present in the body in sufficient quantities to theoretically supply the body with fuel for several days [12]. Half a kilogram of fat provides around 4500 kcal and the high storage capacity makes fat the energy source of choice for low to moderate intensities when enough oxygen is available [19,20,21]. Combined with exercise, a high rate of fat oxidation modulates insulin sensitivity and glucose tolerance. Hence, enhanced fat oxidation is not only a goal for athletes but also for the general population as it might improve performance at first glance and health in the longer-term speaking [22,23].

Substrate utilization can be influenced through the intake of exogenous carbohydrates during exercise. Since the beginning of the 20th century, the effects of CHO intake during prolonged endurance exercise have been of interest for researchers [24,25,26]. Dill, et al. [27] for example, found as early as 1932 that the blood sugar level in dogs could be kept constant during prolonged exercise by administering 20 g of carbohydrates per hour and, furthermore, the dogs could maintain a certain speed for a longer time-period compared to when consuming water. Moreover, it was found that by supplying carbohydrates before and during exercise, symptoms of hypoglycaemia could be avoided and endogenous carbohydrate stores were spared [28,29,30]. Controlled studies have shown that carbohydrate supplementation can prolong performance during endurance exercise because CHO oxidation can be maintained for a longer period of time compared to placebo administration [31,32,33,34]. The understanding that carbohydrates exert an influence on performance was established many years ago. Since then, numerous studies have been conducted to find the optimal amount, type, and timing of carbohydrate intake during exercise [35]. The current recommendations suggest ingesting up to 60 g·h^−1^ of rapidly available carbohydrates such as glucose or glucose-fructose mixes for activities that are not longer than 2.5 h. When mixtures of glucose and fructose are ingested, this amount could be increased up to 90 g·h^−1^ for prolonged exercise, because of different intestinal transport pathways [1,2,36].

**Figure 1:**
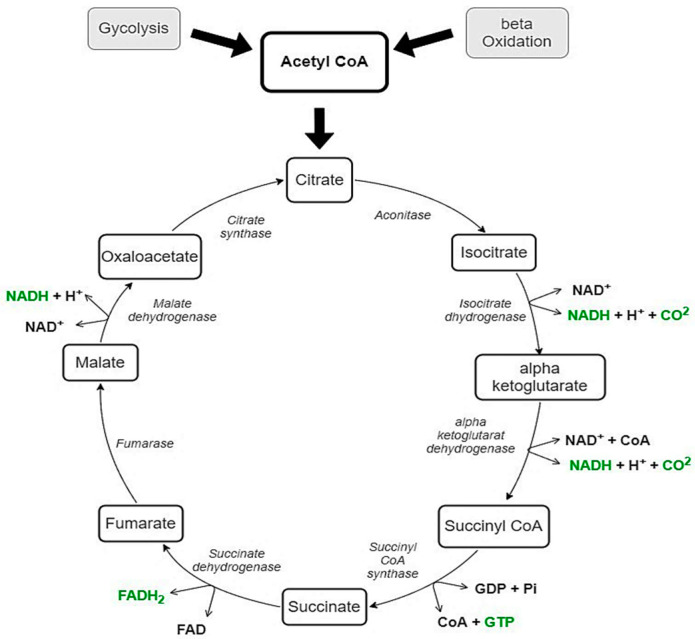
The TCA cycle with all intermediates (black), energy providing products (green), and enzymes (italic). The formation of acetyl CoA is carried out either by glycolysis or beta-oxidation. NADH = nicotinamide adenine dinucleotide; FADH_2_ = flavin adenine dinucleotide; GDP = guanosine diphosphate; GTP = guanosine triphosphate.

As shown in Figure 1, both CHO via glycolysis and fat via beta-oxidation can supply acetyl CoA for the tricarboxylic acid cycle (TCA, also known as the Krebs cycle or citric acid cycle) [37,38]. Therefore, these two mechanisms are closely related. Thus, the mnemonic that fat burns in the fire of carbohydrates was presented. On the one hand, it has been observed that the complete oxidation of fatty acids is facilitated by the simultaneous oxidation of carbohydrates. Early studies have shown that the intermediates from the TCA have an igniting effect on fat oxidation by accumulating adenosine diphosphate (ADP) and forming an acetyl-acceptor in the form of oxaloacetate [38]. On the other hand, however, research with isotopes has shown that carbohydrate metabolism can limit the oxidation of fatty acids by limiting the production of ketone bodies [39]. Early studies conducted on isolated cells have shown that acetyl CoA derived from carbohydrates is accessible for forming acylcarnitine, while acetyl CoA from beta-oxidation is more readily available for the TCA cycle. However, the rate of fatty acid oxidation is influenced by the acetyl CoA derived from the pyruvate metabolism [40]. Because of the steps in the metabolism of carbohydrates and lipids that occur before the TCA cycle, it is difficult to draw conclusions from studies with isolated cells or where only the isolated substrate was added to start the TCA cycle.

When both carbohydrates and fats are present for fuel, the muscle tends to prioritize the oxidation of carbohydrates. This preference for carbohydrates is due to their higher glycolytic flux and the resulting increased production of acetyl CoA. Consequently, carbohydrates hinder the oxidation of fats by inhibiting the production of acetyl CoA from fat and impeding the transport of long-chain fatty acids into the mitochondria [41]. However, the oxidation of fatty acids also regulates the rate and fate of glucose metabolism in the muscle. This reciprocal relationship between the oxidation of the two fuels is known as the glucose/fatty acid cycle [42].

Insulin counts as a key regulator in this complex interplay. It is known as an anabolic hormone, which regulates the storage of energy in the form of glycogen and fat [43]. During the process of energy production, insulin has various effects on fat oxidation. Firstly, insulin stimulates the activity of acetyl-CoA carboxylase (ACC), an enzyme responsible for the conversion of acetyl CoA into malonyl-CoA. Malonyl-CoA, in turn, hinders the function of carnitine palmitoyltransferase (CPT), which is responsible for transporting fatty acids into the mitochondria for energy generation. Monitoring the concentration of malonyl-CoA can provide insights into the availability of carbohydrates as a substrate. When carbohydrates are oxidized (CHO oxidation), there is an increase in glycolytic flow and a greater production of pyruvate. Consequently, the concentration of acetyl CoA, and hence malonyl-CoA, rises, leading to a decrease in fatty acid oxidation. This decrease occurs as CPT’s activity is inhibited, reducing the transportation of long-chain fatty acids into the mitochondria [44,45]. Secondly, insulin is a potent inhibitor of lipolysis, working by reducing non-esterified free fatty acid (NEFA) availability. During the process of fatty acid release from adipose tissue, insulin hinders the activity of hormone-sensitive lipase in adipose tissue. This inhibition leads to a decrease in the levels of non-esterified fatty acids (NEFAs) in the bloodstream. Consequently, the muscle is compelled to utilize glucose to a greater extent. When plasma insulin increases, after the administration of carbohydrates [46,47], a reduction in total fat oxidation could be observed [41,48,49,50]. During periods of low CHO availability, insulin secretion is suffocated and the organism relies on fat as a fuel [43].

Overall, insulin has two significant effects on substrate oxidation, ultimately resulting in the inhibition of fat oxidation and the promotion of glucose utilization: (1) increasing malonyl-CoA concentration through activated ACC and thereby decreasing the rate of fatty acid oxidation via inhibited CPT activity; and (2) reducing NEFA availability via the effect on adipose tissue lipolysis. In the fed state (i.e., when insulin secretion is stimulated), insulin regulates energy storage and limits fat oxidation, whilst in the fasted state, fat oxidation is promoted. 

In summary, since the GI reflects the insulinemic response of a CHO, low-GI foods can attenuate the suppression of fat oxidation compared to high-GI foods. A low-GI CHO results in a lower postprandial increase in blood glucose and insulin and consequently a mitigated inhibition of fat oxidation. 

Modifying substrate metabolism through dietary and exercise interventions has been a subject of interest for several years. For prolonged low-intensity endurance exercise, it is beneficial to promote high fat oxidation as it helps to reduce lactate concentrations and conserve limited carbohydrate stores. Various approaches and dietary plans, such as ketogenic or low-carbohydrate, high-fat diets (LCHF), have been suggested to alter substrate metabolism and, consequently, enhance endurance performance. During a low-carbohydrate, high-fat (LCHF) diet, the intake of carbohydrates (<20 E-% carbohydrates per day, >50 E-% fat per day) is drastically reduced. These metabolic changes facilitate an increase in the availability of free fatty acids, resulting in a reduced utilization of muscle glycogen and a reduction in CHO oxidation during physical activity [16,47,51]. After two to three weeks on a LCHF diet, the body enters a ketogenic state, which is thought to enhance performance during prolonged exercise by improving substrate utilization in favor of fat and conserving muscle glycogen [52,53]. The process of oxidizing non-esterified fatty acids in the liver produces ketone bodies (such as acetoacetate, acetone, etc.), leading to ketosis. In situations when carbohydrate availability is limited, muscles oxidize ketone bodies to generate energy [16,54]. Volek, et al. [55] demonstrated that a long-term LCHF diet can increase maximal fat oxidation to 1.5 g/min and the intensity at which it occurs to 70% VO_2_max. Phinney, Bistrian, Evans, Gervino and Blackburn [53] additionally found that after four months on the LCHF diet, CHO oxidation decreases and muscle glycogen is conserved. The same was observed by McSwiney, et al. [56] After following a ketogenic diet for a week, the maximum rate of fat oxidation increased by a factor of two.

Kang, et al. [57] conducted a systematic review that summarized all the studies examining the effects of a ketogenic diet on substrate metabolism. All studies reported a decrease in respiratory exchange ratio and carbohydrate oxidation in favor of fat oxidation. Therefore, considerable research has been conducted on the effects of a ketogenic diet on substrate metabolism. Nevertheless, the influence of this dietary approach on overall performance, particularly in competitive settings, remains a topic of debate and controversy.

So far, there are mixed results on the influence of LCHF on VO_2_max, a marker for endurance capacity [19]. Among the six studies included in this review, three found an increase in VO_2_max independent of the nutritional intervention [58,59,60], whereas two studies found no difference from pre to post intervention [53,61], and one study did not measure VO_2_max after intervention [62]. Regarding LCHF and its influence on VO_2_max, further data are needed. Possible explanations for changes in VO_2_max include changes in oxidative metabolic processes [59,60], the production of certain metabolic by-products [16], such as tryptophan and ammonium, or reduced energy intake [52]. By promoting an elevation in intramuscular triglycerides, lipase activity, and the expression of fatty acid translocase FAT/CD36 protein and CPT [59], a low-carbohydrate, high-fat (LCHF) diet can potentially enhance performance at the aerobic threshold [63,64,65].

However, as far as competitive endurance performance is concerned, a high-fat diet could even have a negative impact on performance, particularly at higher intensities. This occurs due to the low ability of the muscles to efficiently activate glycogenolysis and pyruvate dehydrogenase due to the absence of sufficient carbohydrates during prior training or exercise sessions. [66]. An ergogenic effect of a LCHF diet could therefore not be convincingly proven, as substrate deficits are present especially in the high-intensity range and thus training optimizations are more difficult [67].

### 1.2. GI and Its Relevance in Sport Nutrition

**Figure 2:**
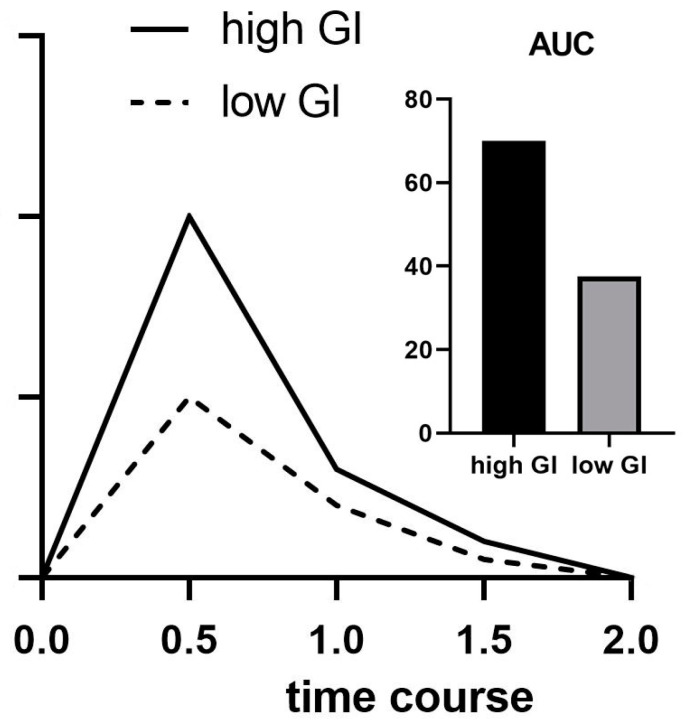
Schematic presentation of high- and low-GI foods and their respective area under the curve (AUC).

The GI indicates the impact that a carbohydrate has on blood glucose levels. The GI can therefore be seen as an indicator of how quickly or slowly the body digests and absorbs the CHO present in the food consumed [68]. The GI is calculated by measuring the area under the curve (AUC) of blood glucose for 2 h after consumption of the test food, containing 50 g of available carbohydrates and a reference food that also contains 50 g of available carbohydrates [69]. The AUC of the test food is then compared to the reference food. The GI is expressed as a percentage of the quotient of AUC from the tested foods (Figure 2) [4]. Foods can then be classified based upon their GI. A list of GI values of common foods can be found at Atkinson, et al. [70].

The importance of the GI has increased steadily in recent years. In the last 12 years, the number of scientific publications involving the GI or glycaemic load has tripled, from about 2500 to 7500 [71]. There are still concerns about the variation in published GI values for apparently identical foods. These variations derive either from methodological issues or differences in the physical and chemical characteristics of the food. This is also the reason why the GI cannot be calculated and must be measured. The exact composition of the ingredients and the processing can have an important influence on the rate of CHO digestion and hence the GI. Furthermore, different testing methods may cause variations in GI values [72]. Nevertheless, published GI values show the mean of the reported values from a number of studies conducted in different laboratories [70].

Since the GI indicates how quickly a CHO affects blood glucose, it seems reasonable that eating foods with different GI values before, during, and after exercise has an impact on athletic performance [68]. As the concept of the GI in sports is relatively new, there are still many unanswered questions regarding its role in sport nutrition [73].

In particular, the interest in low GI carbohydrates and their effects in athletic performance and substrate metabolism has increased in recent years. The slow absorption and gradual release of these carbohydrates provide a steady supply of energy during prolonged exercise and promote fat oxidation [7,8,74,75]. Consequently, because low GI carbohydrates result in a reduced blood glucose and insulin response, they help maintain normal blood sugar levels (i.e., euglycemia) and preserve muscle glycogen, which in turn affects the utilization of substrates during exercise [76]. The acute effects of low-GI carbohydrates in endurance performance have already been investigated in some studies. For example, in Achten, Jentjens, Brouns and Jeukendrup [74], supplementation with isomaltulose, a low-GI sugar, was most likely to increase fat oxidation compared to sucrose, based on measured differences in plasma insulin. The same observations were made by Oosthuyse, Carstens and Millen [8]. When isomaltulose was administered before exercise, blood glucose levels were more stable during exercise, and fat oxidation was higher [7,77]. Miyashita, et al. [78] also found that taking isomaltulose before exercise resulted in a lower postprandial glucose concentration. By affecting blood glucose and insulin, the GI shifts substrate metabolism towards higher fat oxidation [77,79,80,81,82,83,84,85], even though not all studies have demonstrated this [86,87].

Moreover, there have already been numerous studies examining the impact of consuming a low-glycemic-index (GI) meal before training on performance during the training session. Burdon, Spronk, Cheng and O’Connor [88] conducted a systematic review with meta-analysis of these studies and concluded that consuming a low-GI meal does not result in a significant improvement in endurance performance. However, they did observe a small, non-significant increase in performance when no exogenous carbohydrates were provided during exercise, which may be attributed to the maintenance of carbohydrate availability. Another meta-analysis showed a weak, but positive, effect of a low-GI meal on subsequent endurance exercise [89]. Inconsistent findings may also derive from methodological differences (e.g., meal content and GI, timing, amount of carbohydrates, exercise protocol, …) between studies. In terms of substrate metabolism, a low-GI compared to a high-GI meal before exercise leads to a reduced blood glucose and insulin response [6,83], which could help increase fat oxidation and maintain euglycemia during exercise [90].

Consequently, there have been some observations regarding the immediate effects of glycaemic index on endurance performance. However, thus far, only a limited number of studies have specifically addressed the question of how a long-term diet consisting of carbohydrates with varying GI values affects endurance performance or substrate metabolism.

## 2. Methods

For the following narrative review, the terms “glycaemic index” and “endurance” were used. The databases PubMed, Scopus and Google Scholar were searched up until May 2021.

The retrieved studies were collected and sorted by date and exercise type. Double entries were removed. This review only includes randomized controlled trials. Animal, experimental model studies, or proposed study designs were excluded from the review. Inclusion criteria were: an intervention period lasting at least three days and the administration of two different nutritional interventions differing in GI. Only studies examining endurance athletes were included. A total of seven studies have been included in this narrative review.

## 3. Discussion

Studies investigating the acute effects of glycemic index on endurance performance have not always yielded consistent outcomes. Considering the metabolic pathways outlined earlier, it is reasonable to speculate that the GI affects substrate metabolism during endurance exercise by reflecting the postprandial increase in blood glucose and subsequent insulin secretion.

To our knowledge, the first study that investigated the influence of low- or high-GI meals lasting several days on substrate metabolism and performance was conducted by Chen, et al. [91] and lasted three days. Nine male runners completed a three-day CHO loading with different GI and glycaemic loads in a counterbalanced crossover design. The high-GI as well as the low-GI diet provided 10 g·kg body weight^−1^ of available CHO per day with a GI of 80 and 36, respectively. In order to reduce muscle glycogen before the intervention, the participants completed a 1 h exhausting exercise consisting of a 30 min run at 80% of VO_2_max followed by 30 min at 70% of VO_2_max. After following the three days of standardized diet, the runners had to run 1h at 70% of VO_2_max, followed by a 10 km time trial. No difference in fat or carbohydrate oxidation nor in the completion of the 10 km time trial was observed between the high- and low-GI trials (Table 1). Further, results from Hamzah, et al. [92] indicated that substrate metabolism during a running exercise in a fasted state is not influenced by GI. In a randomized counterbalanced order, each of the nine subjects completed a five-day low- or high-GI diet. After the five days, subjects ran until exhaustion on a treadmill at a speed equivalent to 65% of VO_2_max. No difference in plasma insulin at the timepoint of exhaustion was observed between the trials. During the run to exhaustion, the rate of fat oxidation was significantly lower in the CHO trials compared to the control trial. Subsequently, CHO oxidation during exercise was higher in the CHO trials compared to control. At the timepoint of exhaustion, the rate of fat oxidation was lower, and consequently, the oxidation of carbohydrates was higher only in the low-GI trial compared to the control. Neither time to exhaustion nor distance covered were different between the trials (Table 1). Hamzah, Higgins, Abraham, Taylor, Vizbaraite and Malkova [92] concluded that GI does not reduce fat oxidation during running in a fasted state. In addition, GI has no impact on running capacity (Table 1). In a 5-day study conducted by Cocate, Pereira, Marins, Cecon, Bressan and Alfenas [6], with 15 male cyclists, a lower glycaemic and insulinemic response was observed with a low-GI test meal compared to a high-GI test meal. However, during an exercise test, no differences were found between groups. The GI had no impact on free fatty acids levels before and during exercise or on substrate metabolism during exercise. However, the fat oxidation 90 min after ingestion was higher in the high-GI group compared to low-GI group (Table 1), which might be explained by the lower fructose content in the low-GI meal. Fructose counts as a low-GI sugar, and it has been shown that the consumption of 50 g fructose leads to lower fat oxidation compared to glucose [93]. Nonetheless, these results indicate that when consuming a high CHO diet in preparation for an endurance event, the consideration of GI is not necessary. The consumption of high CHO diets might lead to the reduced oxidation of fat because of a reduced availability and utilization of intramyocellular lipids (IMCT) [94,95].

Therefore, when considering the glycemic index over a span of several days, it is unlikely that merely lowering the GI of consumed foods will achieve a significant increase in fat oxidation and a consequential preservation of muscle glycogen. What has been observed, however, is that a CHO-rich diet based on a low GI is able to maintain CHO availability longer than the control diet, as blood glucose levels and carbohydrate oxidation at the time of exhaustion tended to be higher in the low-GI group compared to the control group. This contrasts with the results of Chen, Wong, Xu, Hao, Wong and Lam [91], who found no difference in blood glucose between the two CHO groups after the 10 km time trial. However, sample sizes in these studies were small. Additionally, the rather short intervention periods of three and five days might not be sufficient to draw clear conclusions about the chronic metabolic effects of GI.

It is plausible that longer-term low-GI diets (intervention periods longer than one week) can result in more substantial changes in substrate metabolism compared to short-term interventions. These diets may lead to increased fat oxidation due to reduced carbohydrate (CHO) availability during exercise and lower postprandial insulin levels. Additionally, it is hypothesized that the enhanced availability of non-esterified fatty acids could potentially enhance the activity of mitochondrial enzymes [96]. In a study conducted by Durkalec-Michalski, et al. [97] a total of 21 runners followed a moderate- or low-GI diet in a crossover design for three weeks. A carbohydrate-rich diet with a low-GI has been demonstrated to enhance performance in a 12 min running test and prolong time to exhaustion in an incremental cycling test. However, the extent of improvement did not exhibit a significant difference when compared to a diet with a medium GI. Regarding substrate oxidation, the authors made assumptions from the measured breathing gases during the incremental cycling test. Both diets were able to increase intensity and power at the gas exchange threshold (GET) (Table 1). It is speculated that the GET is correlated with maximal fat oxidation and the point where fat utilization becomes unimportant. Therefore, the later occurrence of the GET and the higher intensity might be correlated to a better fat oxidation capacity [97]. Another study conducted by the same research team focused on investigating the effects of low and moderate glycemic index diets on substrate metabolism. The researchers measured substrate metabolism by analyzing the gases inspired and expired during exercise. In summary, from the data presented, no significant metabolic changes between groups could be observed. Regardless of GI, both nutritional regimes resulted in a slight decrease in fat oxidation during the incremental cycling test. The moderate-GI diet resulted in increased CHO oxidation and improved performance, which was assessed as time to exhaustion in the incremental cycling test (Table 1) [98].

Given that the objective of a low-GI diet is to induce favorable metabolic adaptations, such as increased fat oxidation and reduced utilization of muscle glycogen, it is reasonable to compare this dietary approach with a low-carbohydrate, high-fat (LCHF) diet, in which these adaptations have already been observed. The advantage of a low-GI diet over an LCHF diet is that performance at higher intensities is not reduced because carbohydrates are present, and the organism maintains the metabolic pathways (namely the enzymes glycogen phosphorylase, phosphofructokinase, and pyruvate dehydrogenase) to utilize them. In a four-week trial, Zdzieblik, et al. [99] examined the effects of three different diets with varying amounts and types of CHO on metabolic processes under resting and exercise conditions in 28 male endurance athletes. Assumptions regarding fat and carbohydrate metabolism were made based on lactate concentrations in the incremental test. In the low-GI group, lactate concentrations under resting conditions and in submaximal test settings were significantly lower compared to baseline. Therefore, a possible positive influence of a low-GI diet on fat oxidation was assumed. A decreased RER value under resting conditions and during submaximal exercise in the LCHF group, combined with lower lactate concentrations, indicated increased fat oxidation. However, it must also be stated that a significant decrease in time to exhaustion during the incremental test was only observed in the LCHF group, whereas no changes were present in the CHO groups. These adaptations are an argument for the low-GI diet, as CHO metabolism remained unaffected at higher intensities while fat oxidation was improved with submaximal exercise. The LCHF group failed to improve at higher intensities, presumably because carbohydrates were missing as a substrate. During prolonged strenuous exercise, the metabolic flexibility induced by low-GI carbohydrates could provide benefits. Taking a closer look at the performance outcomes of the incremental test, a significant difference between the three intervention groups was observed in absolute and relative peak power output. After four weeks, the LCHF diet resulted in a reduction in absolute peak power and VO_2_max, whereas no significant changes were observed in the CHO groups. Nonetheless, power at the lactate threshold significantly improved in the LCHF group (Table 1) [100]. As already stated, a LCHF diet might be beneficial for enhancing performance at submaximal intensities, but it hinders adaption at high exercise intensity. On the contrary, a low-GI diet seems to be favorable for enriched performance at both—moderate and severe—intensities due to enhanced metabolic flexibility.

**Table 1:** Summary of studies.

	Study Design	GI of Diets	Duration	Subjects	Results	
					Performance	Substrate Metabolism
Chen et al., 2008 [91]	crossover	high GI: 80 low GI: 36	3 days	9 male runners (VO_2_max: 61.9 ± 2.9 mL·min^−1^·kg^−1^)	no difference in 10 km TT (high GI: 51.3 ± 5.3 vs. Low GI: 48.6 ± 1.3 min)	no difference in CHO or fat oxidation before, during or after trials
Hamzah et al., 2009 [92]	crossover	high GI: 71 ± 1 low GI: 36 ± 0	5 days	9 males (VO_2_max: 59.8 ± 4.3 mL·min^−1^·kg^−1^)	no difference in TTE (high GI: 107 ± 18 vs. low GI: 107 ± 18 min) and distance covered (high GI: 18 ± 5 vs. low GI: 19 ± 5 km)	no differences in CHO or fat oxidation in fasted state, during exercise or at exhaustion
Cocate et al., 2011 [6]	crossover	high GI: 79 low GI: 28	5 days	15 male cyclists (VO_2_max: 70.0 ± 5.3 mL·min^−1^·kg^−1^)		higher postprandial fat oxidation in high GI compared to low GI higher postprandial CHO oxidation in low GI compared to high GI
Durkalec-Michalski et al., 2018 [97]	crossover	moderate GI low GI	21 days	10 male and 7 female runners (VO_2_max: 50.5 ± 7.4 mL·min^−1^·kg^−1^)	longer TTE in moderate GI (12:58 ± 3:31) vs. low GI (12:32 ± 3:44)	no differences in CHO of fat oxidation
Durkalec-Michalski et al., 2018 [98]	crossover	moderate GI: 61 ± 1 low GI: 39 ± 1	21 days	13 males and 7 female runners (VO_2_max: 51.6 ± 9.4 mL·min^−1^·kg^−1^)	no differences in 12 min running test or TTE in ICT	
Zdzieblik et al., 2022 [100]	parallel groups	high GI: 74 ± 3 low GI: 39 ± 4 LCHF	28 days	28 males	no differences in TTE or lactate concentration at ICT in CHO groups	
Zdzieblik et al., 2022 [99]	parallel groups	high GI: 74 ± 3 low GI: 39 ± 4 LCHF	28 days	28 males		higher fat oxidation in LCHF due to lower lactate concentration compared to baseline in low GI an improved fat oxidation was assumed

GI = glycaemic index; LCHF = low-carb high-fat diet; TT = time trial; TTE = time to exhaustion; ICT = incremental cycling test.

## 4. Research Gaps

The current body of research on the longer-term effects of a low-glycemic-index diet on athletic performance and substrate metabolism is limited, and there exist conflicting findings. However, it can be assumed that the duration of the intervention plays a crucial role in establishing the positive effects of a low-GI diet during regular training. A significant challenge in studies with longer-term nutritional interventions is ensuring subjects’ compliance with their assigned diets. Moreover, larger sample sizes are necessary to draw more-precise and reliable conclusions.

In addition, the precise mechanisms behind the metabolic adaptations following a longer-term low-GI diet are still not clear. As stated earlier, insulin and its effects on substrate utilization influence metabolic processes and substrate use. In order to gain further clarity on the effects of a low-GI diet on athletic performance and substrate metabolism, additional measurements of muscular energy stores, as well as the assessment of hormones and enzymes involved in substrate metabolism, would be valuable. These additional measurements can provide more comprehensive insights and help shed light on the underlying mechanisms and specific metabolic adaptations associated with a low-GI diet.

In addition, the use of metabolomics could help to further investigate the complex and interrelated interplay between physical activity and nutrition. Every meal, and also exercise, changes the metabolism, and there are more than 150 associated and identified metabolites with this interplay [101]. These measurements can precisely detect metabolites and their fragments involved in energy supply and therefore provide a better understanding of metabolic adaptations happening through a low-GI diet. 

In summary, to provide more accurate insights into the longer-term effects of a low-GI diet on substrate metabolism and endurance performance, the following questions need to be addressed:

- Duration of diet: How long should a low-GI diet be maintained to sustainably change substrate metabolism?
- Mechanistic effects: What are the underlying mechanisms through which a low-GI diet affects muscular energy stores and metabolism?
- Comparisons to an LCHF diet: Can a long-term low-GI diet achieve similar adaptations in fat metabolism to an LCHF diet without compromising performance at higher intensities and impairing carbohydrate metabolism?

Answering these questions would provide a more comprehensive understanding of the effects of a low-GI diet on endurance performance and substrate metabolism, allowing for more accurate statements and recommendations.

## 5. Conclusions

The objective of this review was to provide an overview of the existing knowledge regarding the potential longer-term influence of the GI on substrate utilization during exercise. The main focus was to examine the available literature and determine whether the consumption of a long-term high-carbohydrate diet with different glycaemic indices could have an impact on substrate metabolism and endurance performance. At present, there is still limited evidence available regarding the effects of a long-term high-carbohydrate diet with a low GI on substrate metabolism or performance. Specifically, although the physiological background is sound and some studies have found supporting results, the notion that diets featuring low-glycaemic-index carbohydrates enhance performance by promoting fat oxidation and preserving glycogen stores lacks convincing evidence from large studies. Nevertheless, further research is necessary to explore the potential effects observed in cellular or substrate studies, particularly with respect to the influence of high glucose and insulin levels on metabolic pathways related to fat oxidation. In summary, data so far suggest, but do not prove, that a low-GI diet could enhance metabolic flexibility and consequently enhance endurance performance by influencing substrate metabolism (e.g., through the longer-term alteration of insulin secretion). In order to provide practical recommendations for endurance athletes, rather than completely eliminate carbohydrates (as in a low-carbohydrate diet), athletes could be cautiously advised to incorporate specific low-GI carbohydrates into their diet. This approach would help maintain performance at high intensities while simultaneously promoting fat oxidation, as compared to a high-GI diet.

## Data Availability

Not applicable.

## References

[1] Thomas D., Burke L., Erdman K. (2016). Nutrition and Athletic Performance. Med. Sci..

[2] Podlogar T., Wallis G.A. (2022). New Horizons in Carbohydrate Research and Application for Endurance Athletes. Sport. Med..

[3] Jenkins D.J., Wolever T.M., Taylor R.H., Barker H., Fielden H., Baldwin J.M., Bowling A.C., Newman H.C., Jenkins A.L., Goff D.V. (1981). Glycemic index of foods: A physiological basis for carbohydrate exchange. Am. J. Clin. Nutr..

[4] Wolever T.M., Jenkins D.J., Jenkins A.L., Josse R.G. (1991). The glycemic index: Methodology and clinical implications. Am. J. Clin. Nutr..

[5] Carneiro L., Leloup C. (2020). Mens sana in corpore sano: Does the Glycemic Index Have a Role to Play?. Nutrients.

[6] Cocate P.G., Pereira L.G., Marins J.C., Cecon P.R., Bressan J., Alfenas R.C. (2011). Metabolic responses to high glycemic index and low glycemic index meals: A controlled crossover clinical trial. Nutr. J..

[7] König D., Zdzieblik D., Holz A., Theis S., Gollhofer A. (2016). Substrate Utilization and Cycling Performance Following Palatinose™ Ingestion: A Randomized, Double-Blind, Controlled Trial. Nutrients.

[8] Oosthuyse T., Carstens M., Millen A.M. (2015). Ingesting Isomaltulose Versus Fructose-Maltodextrin During Prolonged Moderate-Heavy Exercise Increases Fat Oxidation but Impairs Gastrointestinal Comfort and Cycling Performance. Int. J. Sport Nutr. Exerc. Metab..

[9] Burke L.M. (2021). Ketogenic low-CHO, high-fat diet: The future of elite endurance sport?. J. Physiol..

[10] Burke L.M. (2015). Re-Examining High-Fat Diets for Sports Performance: Did We Call the ‘Nail in the Coffin’ Too Soon?. Sport. Med..

[11] Howard E.E., Margolis L.M. (2020). Intramuscular Mechanisms Mediating Adaptation to Low-Carbohydrate, High-Fat Diets during Exercise Training. Nutrients.

[12] Alghannam A.F., Ghaith M.M., Alhussain M.H. (2021). Regulation of Energy Substrate Metabolism in Endurance Exercise. Int. J. Environ. Res. Public Health.

[13] Spriet L.L. (2014). New Insights into the Interaction of Carbohydrate and Fat Metabolism During Exercise. Sport. Med..

[14] Hargreaves M., Spriet L.L. (2018). Exercise Metabolism: Fuels for the Fire. Cold Spring Harb. Perspect. Med..

[15] Romijn J.A., Coyle E.F., Sidossis L.S., Gastaldelli A., Horowitz J.F., Endert E., Wolfe R.R. (1993). Regulation of endogenous fat and carbohydrate metabolism in relation to exercise intensity and duration. Am. J. Physiol..

[16] Chang C.-K., Borer K., Lin P.-J. (2017). Low-Carbohydrate-High-Fat Diet: Can it Help Exercise Performance?. J. Hum. Kinet..

[17] Venables M.C., Achten J., Jeukendrup A.E. (2005). Determinants of fat oxidation during exercise in healthy men and women: A cross-sectional study. J. Appl. Physiol..

[18] Ormsbee M., Bach C., Baur D. (2014). Pre-Exercise Nutrition: The Role of Macronutrients, Modified Starches and Supplements on Metabolism and Endurance Performance. Nutrients.

[19] Bailey C.P., Hennessy E. (2020). A review of the ketogenic diet for endurance athletes: Performance enhancer or placebo effect?. J. Int. Soc. Sport. Nutr..

[20] Jeukendrup A., Saris W., Wagenmakers A. (1998). Fat Metabolism During Exercise: A Review—Part II: Regulation of Metabolism and the Effects of Training. Int. J. Sport. Med..

[21] Jeukendrup A., Saris W., Wagenmakers A. (1998). Fat Metabolism During Exercise: A Review. Part I: Fatty Acid Mobilization and Muscle Metabolism. Int. J. Sport. Med..

[22] Bonen A., Dohm G.L., van Loon L.J. (2006). Lipid metabolism, exercise and insulin action. Essays Biochem..

[23] Ni C., Jia Q., Ding G., Wu X., Yang M. (2022). Low-Glycemic Index Diets as an Intervention in Metabolic Diseases: A Systematic Review and Meta-Analysis. Nutrients.

[24] Christensen E.H., Hansen O. (1939). II. Hypoglykämie, Arbeitsfähigkeit und Ermüdung. Skand. Arch. Für Physiol..

[25] Christensen E.H., Hansen O. (1939). III. Arbeitsfähigkeit und Ernährung. Skand. Arch. Für Physiol..

[26] Krogh A., Lindhard J. (1920). The Relative Value of Fat and Carbohydrate as Sources of Muscular Energy: With Appendices on the Correlation between Standard Metabolism and the Respiratory Quotient during Rest and Work. Biochem. J..

[27] Dill D.B., Edwards H.T., Talbott J.H. (1932). Studies in muscular activity: VII. Factors limiting the capacity for work. J. Physiol..

[28] Gordon B., Kohn L.A., Levine S.A., Matton M., Scriver W.M., Whiting W.B. (1925). Sugar content of the blood in runners following a marathon race: With special reference to the prevention of hypoglycemia: Further observations. J. Am. Med. Assoc..

[29] Levine S.A., Grodon B., Derick C.L. (1924). Some changes in the chemical constituents of the blood following a marathon race: With special reference to the development of hypoglycemia. J. Am. Med. Assoc..

[30] McConell G., Snow R.J., Proietto J., Hargreaves M. (1999). Muscle metabolism during prolonged exercise in humans: Influence of carbohydrate availability. J. Appl. Physiol..

[31] Febbraio M.A., Chiu A., Angus D.J., Arkinstall M.J., Hawley J.A. (2000). Effects of carbohydrate ingestion before and during exercise on glucose kinetics and performance. J. Appl. Physiol..

[32] Wallis G.A., Yeo S.E., Blannin A.K., Jeukendrup A.E. (2007). Dose-response effects of ingested carbohydrate on exercise metabolism in women. Med. Sci. Sport. Exerc..

[33] Carter J.M., Jeukendrup A.E., Mann C.H., Jones D.A. (2004). The Effect of Glucose Infusion on Glucose Kinetics during a 1-h Time Trial. Med. Sci. Sport. Exerc..

[34] Wilber R.L., Moffatt R.J. (1992). Influence of carbohydrate ingestion on blood glucose and performance in runners. Int. J. Sport Nutr..

[35] Jeukendrup A.E. (2010). Carbohydrate and exercise performance: The role of multiple transportable carbohydrates. Curr. Opin. Clin. Nutr. Metab. Care.

[36] Vitale K., Getzin A. (2019). Nutrition and Supplement Update for the Endurance Athlete: Review and Recommendations. Nutrients.

[37] Akram M. (2014). Citric Acid Cycle and Role of its Intermediates in Metabolism. Cell Biochem. Biophys..

[38] Bowtell J.L., Marwood S., Bruce M., Constantin-Teodosiu D., Greenhaff P.L. (2007). Tricarboxylic Acid Cycle Intermediate Pool Size. Sport. Med..

[39] Masoro E.J., Felts J.M. (1958). Role of carbohydrate metabolism in promoting fatty acid oxidation. J. Biol. Chem..

[40] Abdel-aleem S., Nada M.A., Sayed-Ahmed M., Hendrickson S.C., St Louis J., Walthall H.P., Lowe J.E. (1996). Regulation of fatty acid oxidation by acetyl-CoA generated from glucose utilization in isolated myocytes. J. Mol. Cell. Cardiol..

[41] Coyle E.F., Jeukendrup A.E., Wagenmakers A.J., Saris W.H. (1997). Fatty acid oxidation is directly regulated by carbohydrate metabolism during exercise. Am. J. Physiol..

[42] Randle P.J. (1998). Regulatory interactions between lipids and carbohydrates: The glucose fatty acid cycle after 35 years. Diabetes Metab Rev..

[43] Dimitriadis G., Mitrou P., Lambadiari V., Maratou E., Raptis S.A. (2011). Insulin effects in muscle and adipose tissue. Diabetes Res. Clin. Pr..

[44] Odland L.M., Heigenhauser G.J., Lopaschuk G.D., Spriet L.L. (1996). Human skeletal muscle malonyl-CoA at rest and during prolonged submaximal exercise. Am. J. Physiol..

[45] McGarry J.D., Mannaerts G.P., Foster D.W. (1977). A possible role for malonyl-CoA in the regulation of hepatic fatty acid oxidation and ketogenesis. J. Clin. Investig..

[46] DeFronzo R.A., Ferrannini E., Hendler R., Felig P., Wahren J. (1983). Regulation of splanchnic and peripheral glucose uptake by insulin and hyperglycemia in man. Diabetes.

[47] Costill D.L., Coyle E., Dalsky G., Evans W., Fink W., Hoopes D. (1977). Effects of elevated plasma FFA and insulin on muscle glycogen usage during exercise. J. Appl. Physiol. Respir. Environ. Exerc. Physiol..

[48] Campbell P.J., Carlson M.G., Hill J.O., Nurjhan N. (1992). Regulation of free fatty acid metabolism by insulin in humans: Role of lipolysis and reesterification. Am. J. Physiol..

[49] Ahlborg G., Felig P. (1976). Influence of glucose ingestion on fuel-hormone response during prolonged exercise. J. Appl. Physiol..

[50] Horowitz J.F., Mora-Rodriguez R., Byerley L.O., Coyle E.F. (1997). Lipolytic suppression following carbohydrate ingestion limits fat oxidation during exercise. Am. J. Physiol..

[51] Vukovich M.D., Costill D.L., Hickey M.S., Trappe S.W., Cole K.J., Fink W.J. (1993). Effect of fat emulsion infusion and fat feeding on muscle glycogen utilization during cycle exercise. J. Appl. Physiol. (1985).

[52] Paoli A., Bosco G., Camporesi E.M., Mangar D. (2015). Ketosis, ketogenic diet and food intake control: A complex relationship. Front. Psychol..

[53] Phinney S.D., Bistrian B.R., Evans W.J., Gervino E., Blackburn G.L. (1983). The human metabolic response to chronic ketosis without caloric restriction: Preservation of submaximal exercise capability with reduced carbohydrate oxidation. Metabolism.

[54] Devrim-Lanpir A., Hill L., Knechtle B. (2021). Efficacy of Popular Diets Applied by Endurance Athletes on Sports Performance: Beneficial or Detrimental? A Narrative Review. Nutrients.

[55] Volek J.S., Freidenreich D.J., Saenz C., Kunces L.J., Creighton B.C., Bartley J.M., Davitt P.M., Munoz C.X., Anderson J.M., Maresh C.M. (2016). Metabolic characteristics of keto-adapted ultra-endurance runners. Metabolism.

[56] McSwiney F.T., Fusco B., McCabe L., Lombard A., Crowley P., Walsh J., Hone M., Egan B. (2021). Changes in body composition and substrate utilization after a short-term ketogenic diet in endurance-trained males. Biol. Sport.

[57] Kang J., Ratamess N.A., Faigenbaum A.D., Bush J.A. (2020). Ergogenic Properties of Ketogenic Diets in Normal-Weight Individuals: A Systematic Review. J. Am. Coll. Nutr..

[58] Carr A., Sharma A., Ross M., Welvaert M., Slater G., Burke L. (2018). Chronic Ketogenic Low Carbohydrate High Fat Diet Has Minimal Effects on Acid–Base Status in Elite Athletes. Nutrients.

[59] Burke L.M., Ross M.L., Garvican-Lewis L.A., Welvaert M., Heikura I.A., Forbes S.G., Mirtschin J.G., Cato L.E., Strobel N., Sharma A.P. (2017). Low carbohydrate, high fat diet impairs exercise economy and negates the performance benefit from intensified training in elite race walkers. J. Physiol..

[60] McSwiney F.T., Wardrop B., Hyde P.N., Lafountain R.A., Volek J.S., Doyle L. (2018). Keto-adaptation enhances exercise performance and body composition responses to training in endurance athletes. Metabolism.

[61] Shaw D.M., Merien F., Braakhuis A., Maunder E.D., Dulson D.K. (2019). Effect of a Ketogenic Diet on Submaximal Exercise Capacity and Efficiency in Runners. Med. Sci. Sport. Exerc..

[62] Heatherly A.J., Killen L.G., Smith A.F., Waldman H.S., Seltmann C.L., Hollingsworth A., O’neal E.K. (2018). Effects of Ad libitum Low-Carbohydrate High-Fat Dieting in Middle-Age Male Runners. Med. Sci. Sport. Exerc..

[63] Helge J.W. (2002). Long-term fat diet adaptation effects on performance, training capacity, and fat utilization. Med. Sci. Sport. Exerc..

[64] Langfort J., Pilis W., Zarzeczny R., Nazar K., Kaciuba-Uściłko H. (1996). Effect of low-carbohydrate-ketogenic diet on metabolic and hormonal responses to graded exercise in men. J. Physiol. Pharmacol..

[65] Zajac A., Poprzecki S., Maszczyk A., Czuba M., Michalczyk M., Zydek G. (2014). The effects of a ketogenic diet on exercise metabolism and physical performance in off-road cyclists. Nutrients.

[66] Hargreaves M., Spriet L.L. (2020). Skeletal muscle energy metabolism during exercise. Nat. Metab..

[67] Murphy N.E., Carrigan C.T., Margolis L.M. (2021). High-Fat Ketogenic Diets and Physical Performance: A Systematic Review. Adv. Nutr..

[68] Donaldson C.M., Perry T.L., Rose M.C. (2010). Glycemic Index and Endurance Performance. Int. J. Sport Nutr. Exerc. Metab..

[69] Jenkins D.J.A., Wolever T.M.S., Jenkins A.L., Thorne M.J., Lee R., Kalmusky J., Reichert R., Wong G.S. (1983). The glycaemic index of foods tested in diabetic patients: A new basis for carbohydrate exchange favouring the use of legumes. Diabetologia.

[70] Atkinson F.S., Foster-Powell K., Brand-Miller J.C. (2008). International tables of glycemic index and glycemic load values: 2008. Diabetes Care.

[71] Atkinson F.S., Brand-Miller J.C., Foster-Powell K., Buyken A.E., Goletzke J. (2021). International tables of glycemic index and glycemic load values 2021: A systematic review. Am. J. Clin. Nutr..

[72] Flavel M., Jois M., Kitchen B. (2021). Potential contributions of the methodology to the variability of glycaemic index of foods. World J. Diabetes.

[73] Thomas D.E., Brotherhood J.R., Brand J.C. (1991). Carbohydrate feeding before exercise: Effect of glycemic index. Int. J. Sport. Med..

[74] Achten J., Jentjens R.L., Brouns F., Jeukendrup A.E. (2007). Exogenous oxidation of isomaltulose is lower than that of sucrose during exercise in men. J. Nutr..

[75] König D., Theis S., Kozianowski G., Berg A. (2012). Postprandial substrate use in overweight subjects with the metabolic syndrome after isomaltulose (Palatinose™) ingestion. Nutrition.

[76] Maresch C.C., Petry S.F., Theis S., Bosy-Westphal A., Linn T. (2017). Low Glycemic Index Prototype Isomaltulose-Update of Clinical Trials. Nutrients.

[77] Wu C.L., Williams C. (2006). A low glycemic index meal before exercise improves endurance running capacity in men. Int. J. Sport Nutr. Exerc. Metab..

[78] Miyashita M., Hamada Y., Fujihira K., Namura S., Sakazaki M., Miyasaka K., Nagai Y. (2019). The effects of isomaltulose ingestion on gastric parameters and cycling performance in young men. J. Exerc. Sci. Fit..

[79] Sun F.-H., O’Reilly J., Li L., Wong S.H.-S. (2013). Effect of the glycemic index of pre-exercise snack bars on substrate utilization during subsequent exercise. Int. J. Food Sci. Nutr..

[80] Kirwan J.P., Cyr-Campbell D., Campbell W.W., Scheiber J., Evans W.J. (2001). Effects of moderate and high glycemic index meals on metabolism and exercise performance. Metabolism.

[81] Sparks M.J., Selig S.S., Febbraio M.A. (1998). Pre-exercise carbohydrate ingestion: Effect of the glycemic index on endurance exercise performance. Med. Sci. Sport. Exerc..

[82] Wee S.L., Williams C., Gray S., Horabin J. (1999). Influence of high and low glycemic index meals on endurance running capacity. Med. Sci. Sport. Exerc..

[83] Stevenson E., Williams C., Nute M. (2005). The influence of the glycaemic index of breakfast and lunch on substrate utilisation during the postprandial periods and subsequent exercise. Br. J. Nutr..

[84] Moore L.J., Midgley A.W., Thurlow S., Thomas G., Mc Naughton L.R. (2010). Effect of the glycaemic index of a pre-exercise meal on metabolism and cycling time trial performance. J. Sci. Med. Sport.

[85] Stevenson E.J., Williams C., Mash L.E., Phillips B., Nute M.L. (2006). Influence of high-carbohydrate mixed meals with different glycemic indexes on substrate utilization during subsequent exercise in women. Am. J. Clin. Nutr..

[86] Backhouse S.H., Williams C., Stevenson E., Nute M. (2007). Effects of the glycemic index of breakfast on metabolic responses to brisk walking in females. Eur. J. Clin. Nutr..

[87] Bennard P., Doucet É. (2006). Acute effects of exercise timing and breakfast meal glycemic index on exercise-induced fat oxidation. Appl. Physiol. Nutr. Metab..

[88] Burdon C.A., Spronk I., Cheng H.L., O’Connor H.T. (2017). Effect of Glycemic Index of a Pre-exercise Meal on Endurance Exercise Performance: A Systematic Review and Meta-analysis. Sport. Med..

[89] Heung-Sang Wong S., Sun F.H., Chen Y.J., Li C., Zhang Y.J., Ya-Jun Huang W. (2017). Effect of pre-exercise carbohydrate diets with high vs low glycemic index on exercise performance: A meta-analysis. Nutr. Rev..

[90] Stannard S.R., Constantini N.W., Miller J.C. (2000). The effect of glycemic index on plasma glucose and lactate levels during incremental exercise. Int. J. Sport Nutr. Exerc. Metab..

[91] Chen Y., Wong S., Xu X., Hao X., Wong C., Lam C. (2008). Effect of CHO Loading Patterns on Running Performance. Int. J. Sport. Med..

[92] Hamzah S., Higgins S., Abraham T., Taylor P., Vizbaraite D., Malkova D. (2009). The effect of glycaemic index of high carbohydrate diets consumed over 5 days on exercise energy metabolism and running capacity in males. J. Sport. Sci..

[93] Tittelbach T.J., Mattes R.D., Gretebeck R.J. (2000). Post-Exercise Substrate Utilization after a High Glucose vs. High Fructose Meal During Negative Energy Balance in the Obese. Obes. Res..

[94] Frayn K.N. (2010). Fat as a fuel: Emerging understanding of the adipose tissue-skeletal muscle axis. Acta Physiol..

[95] Van Loon L.J.L., Greenhaff P.L., Constantin-Teodosiu D., Saris W.H.M., Wagenmakers A.J.M. (2001). The effects of increasing exercise intensity on muscle fuel utilisation in humans. J. Physiol..

[96] Gonzalez J.T., Stevenson E.J. (2012). New perspectives on nutritional interventions to augment lipid utilisation during exercise. Br. J. Nutr..

[97] Durkalec-Michalski K., Zawieja E.E., Zawieja B.E., Podgórski T., Jurkowska D., Jeszka J. (2018). Influence of low versus moderate glycemic index of diet on substrate oxidation and energy expenditure during incremental exercise in endurance athletes: A randomized counterbalanced cross-over trial. Int. J. Food Sci. Nutr..

[98] Durkalec-Michalski K., Zawieja E., Zawieja B., Jurkowska D., Buchowski M., Jeszka J. (2018). Effects of Low Versus Moderate Glycemic Index Diets on Aerobic Capacity in Endurance Runners: Three-Week Randomized Controlled Crossover Trial. Nutrients.

[99] Zdzieblik D., Friesenborg H., Gollhofer A., König D. (2022). Effect of a High Fat Diet vs. High Carbohydrate Diets With Different Glycemic Indices on Metabolic Parameters in Male Endurance Athletes: A Pilot Trial. Front. Nutr..

[100] Zdzieblik D., Friesenborg H., Gollhofer A., König D. (2022). A high carbohydrate diet with a low glycaemic index improves training effects in male endurance athletes. Int. J. Food Sci. Nutr..

[101] Schranner D., Kastenmüller G., Schönfelder M., Römisch-Margl W., Wackerhage H. (2020). Metabolite Concentration Changes in Humans After a Bout of Exercise: A Systematic Review of Exercise Metabolomics Studies. Sport. Med. Open.

